# Antiviral activity of chlorogenic acid against influenza A (H1N1/H3N2) virus and its inhibition of neuraminidase

**DOI:** 10.1038/srep45723

**Published:** 2017-04-10

**Authors:** Yue Ding, Zeyu Cao, Liang Cao, Gang Ding, Zhenzhong Wang, Wei Xiao

**Affiliations:** 1 State Key Laboratory of New-tech for Chinese Medicine Pharmaceutical Process, Jiangsu Kanion Pharmaceutical Co., Ltd., Lianyungang, Jiangsu, China

## Abstract

*Lonicera japonica Thunb*, rich in chlorogenic acid (CHA), is used for viral upper respiratory tract infection treatment caused by influenza virus, parainfluenza virus, and respiratory syncytial virus, ect in China. It was reported that CHA reduced serum hepatitis B virus level and death rate of influenza virus-infected mice. However, the underlying mechanisms of CHA against the influenza A virus have not been fully elucidated. Here, the antiviral effects and potential mechanisms of CHA against influenza A virus were investigated. CHA revealed inhibitory against A/PuertoRico/8/1934(H1N1) (EC_50_ = 44.87 μM), A/Beijing/32/92(H3N2) (EC_50_ = 62.33 μM), and oseltamivir-resistant strains. Time-course analysis showed CHA inhibited influenza virus during the late stage of infectious cycle. Indirect immunofluorescence assay indicated CHA down-regulated the NP protein expression. The inhibition of neuraminidase activity confirmed CHA blocked release of newly formed virus particles from infected cells. Intravenous injection of 100 mg/kg/d CHA possessed effective antiviral activity in mice, conferring 60% and 50% protection from death against H1N1 and H3N2, reducing virus titres and alleviating inflammation in the lungs effectively. These results demonstrate that CHA acts as a neuraminidase blocker to inhibit influenza A virus both in cellular and animal models. Thus, CHA has potential utility in the treatment of the influenza virus infection.

Influenza is an acute viral respiratory illness that causes high morbidity and mortality globally^1^. Three circulating subtypes, type A/H1N1, type A/H3N2 and type B, are well known as influenza viruses that infect humans, causing massive and rapidly evolving global epidemics^2^. A previously unrecognized H7N9 subtype of avian influenza virus which could infect humans was first identified in March 2013 and has caused at least 274 deaths during 3 major epidemic waves in China^3^. In humans, influenza infection of the lower respiratory tract can result in flooding of the alveolar compartment, development of acute respiratory distress syndrome and death from respiratory failure^4^. Cytokine storm during influenza infection is a predictor of morbidity and mortality^5^.

Currently available control measures for influenza include vaccination and two classes of antiviral compounds, the M2 ion channel blockers and the neuraminidase inhibitors (NAIs)^6^. Vaccines must be continually updated to cover currently circulating viral strains, and their protective efficacy is limited in people over 65-year-old who are paradoxically susceptible to influenza^7^. M2 ion channel blockers, with potential neurotoxicity, inhibits influenza A virus only^8^. NAIs act by binding to the active site of the viral NA to prevent release and spread of progeny virions from infected cells during the replication cycle^1^, which is a promising target for anti-influenza drugs screening^9^. However, several strains were reported to be resistant to oseltamivir due to mutations in the viral amino acid sequence^10^,^11^. Resistance and severe respiratory distress syndrome caused by influenza virus have become major public health concerns^7^. Thus, there is an urgent need to develop alternative anti-influenza drugs^12^.

Chlorogenic acid (CHA) (Fig. 1A) is a caffeoylquinic acid component distributed widely in *Lonicera japonica* Thunb, *Crataegus monogyna, Eucalyptus globules*, and *Eupatorium perfoliatum*, and *Vaccinium angustifolium*^13^, as well as several traditional Chinese medicine (TCM) injections^14^,^15^,^16^. CHA has antiviral effects against several viruses, including HIV^17^,^18^, adenovirus^19^, hepatitis B virus^19^, HSVs^20^,^21^, and also inhibits inflammation caused by viral infection^22^. CHA was reported to reduced serum hepatitis B virus level *in vivo*^20^. Results from molecular docking experiments indicated that CHA could be a potential NAI of influenza virus H1N1^23^, H5N1^24^ and H7N9^25^. It was reported that CHA could recover cell viability and increase survival rate in H1N1-infected mice^23^. Previous studies have demonstrated the antiviral effects of CHA against influenza virus H5N1^17^,^26^ and its derivatives against influenza virus H3N2^27^,^28^. However, evidence for the antiviral effects and potential mechanisms is very limited. Moreover, it was administered orally in previous experiments^23^, which could not confirm CHA is an effective component in blood, due to its low oral bioavailability (0.13%)^29^. Thus, the anti-influenza effects should be checked by intravenous injection. On the other hand, it was reported that CHA could down-regulate inflammatory factors in glial cells^30^. However, the action of CHA to alleviate inflammation in lung tissue caused by viral infection also requires careful assessment.

The purpose of this study was to investigate the anti-influenza activity of CHA in MDCK cells and mice by intravenous injection. The mechanism of CHA against the influenza virus was also discussed.

## Results

### Cytotoxic and antiviral activity of CHA on MDCK cells

**Figure 1:**
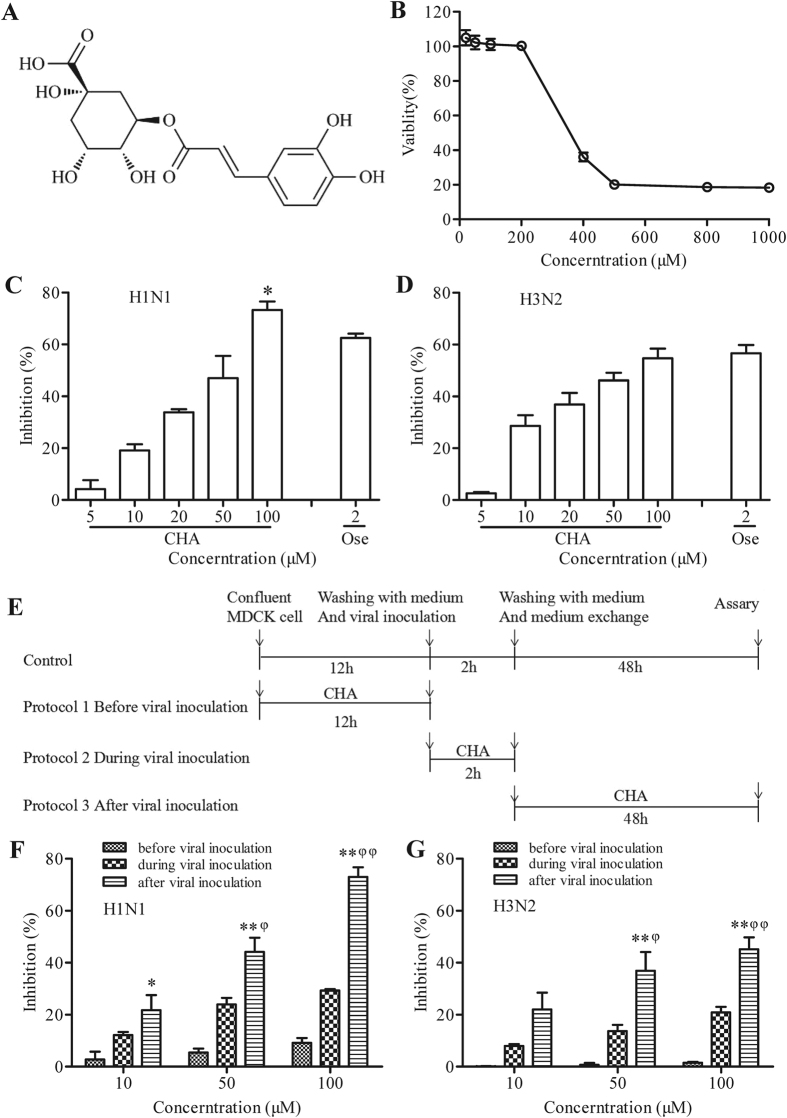
(**A**) Chemical structure of CHA. (**B**) Cellular toxicity of CHA. Inhibitory effects of CHA on (**C**) influenza A/PuertoRico/8/1934(H1N1) and (**D**) A/Beijing/32/92 (H3N2) viruses infection in MDCK cells. *P < 0.05 compared to oseltamivir carboxylate-treated group. (**E**) Experimental protocols to identify the inhibitory effects of CHA on influenza virus infection. MDCK cells (5 × 10^4^ cells/well) were treated with different amounts of CHA, before, at the same time of, and after inoculation with (**F**) influenza A/PuertoRico/8/1934 (H1N1) and (**G**) A/Beijing/32/92 (H3N2) viruses. *P < 0.05, **P < 0.01 compared to Protocol 1; ^φ^*P* < 0.05, ^φφ^*P* < 0.01 compared to Protocol 2.

At a concentration of 200 μM or less, CHA caused no significant cytotoxicity in MDCK cells (Fig. 1B). However, CHA at concentrations over 200 μM reduced the viability of MDCK cells remarkably. The CC_50_ of CHA on MDCK cells was 364.30 ± 1.06 μM.

Inhibitory effects of CHA on the influenza A/PuertoRico/8/1934(H1N1) and A/Beijing/32/92 (H3N2) viruses were first examined *in vitro*. Microscopic examination showed that MDCK cells infected with influenza virus exhibited cytopathic effect (CPE) including cell rounding, detachment and death. Treatment with 10–100 μM CHA significantly reduced CPE caused by infection in MDCK cells. MTS assays revealed that CHA increased the viability of virus-infected cells dose-dependently (Fig. 1C, D). For instance, 100 μM CHA inhibited H1N1 and H3N2 by 73.33% and 54.72%, respectively. Interestingly, 100 μM CHA showed a better antiviral effect than oseltamivir carboxylate (*P* < 0.05), used as positive control, against H1N1. The EC_50_ values were 44.87 ± 1.12 μM and 62.33 ± 1.22 μM against H1N1 and H3N2, respectively (Table 1). Additionally, CHA revealed inhibition against several strains resistant to oseltamivir clinically (Table 2). Moreover, CHA suppressed viral mRNA transcription and subsequent protein translation during H1N1 infection (Suppl. Fig. S1), which confirmed the antiviral effects observed in cellular model. These results indicated that CHA protected MDCK cells from viral infection and reduced the viral production in a dose-dependent manner.

### Inhibitory effects of CHA on different stages of viral replication

To determine the stages by which CHA acts during the influenza virus life cycle, a time-of-addition experiment was conducted following the scheme illustrated in Fig 1E. A less protective effect was observed when CHA was added before viral adsorption, suggesting that the possible target of CHA was rarely located in cell surface. The viability of infected cells was partly recovered by CHA presence during viral adsorption. Moreover, CHA showed even greater inhibition rates by approximately 73.07% and 45.17% against H1N1 and H3N2, respectively, when added after infection (Fig. 1F,G). These results indicated that CHA inhibited a post-adsorption step of the influenza virus life cycle.

### Effects of CHA on the reduction of the viral NP protein

**Figure 2:**
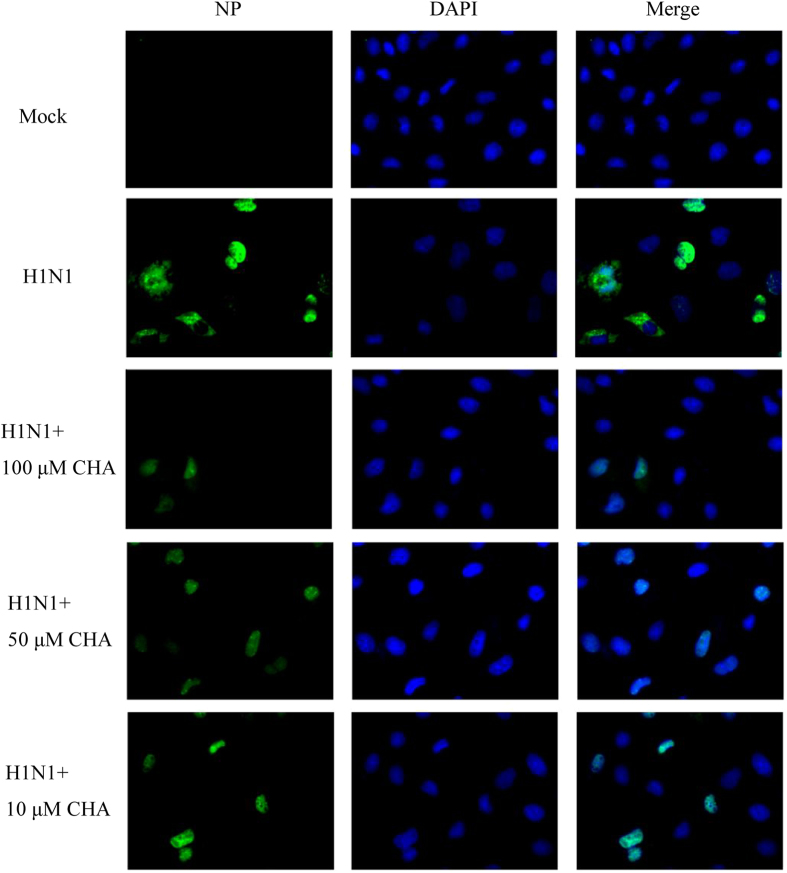
Effects of CHA on viral NP expression level. MDCK cells were infected with A/PuertoRico/8/1934(H1N1), after removing influenza virus and washing with PBS, the cells were incubated with several concentrations of CHA (10, 50, 100 μM) and stained for influenza A virus NP at 24 h pi. (green). The cell nuclei were stained by DAPI (blue). Samples were visualized under a fluorescent microscope.

NP localization was examined at 24 h post infection (pi) when viral replication and transcription was underway and the newly formed virus particles began to release and spread from infected cells. As shown in Fig. 2, no immunofluorescent foci of viral NP was observed in the control. A strong green fluorescent signal was observed in the majority of virus-infected cells without CHA treatment. Moreover, viral NP was localized predominantly in the cytoplasm, with lesser amounts in the nucleus. In contrast, in CHA-treated cells, less NP was observed in the cytoplasm and expression levels decreased dose-dependently (Fig. 2). These results suggested that CHA reduced the expression of viral NP protein and caused nuclear retention of the viral NP, leading to the preventing on the assembly of virus particles.

### Inhibitory effect of CHA on the NA activity of influenza viruses

**Figure 3:**
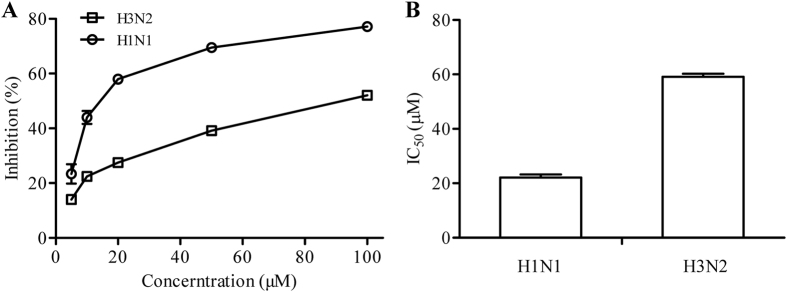
Inhibition of NA activity by CHA. (**A**) Inhibition of the influenza A/PuertoRico/8/1934 (H1N1) and A/Beijing/32/92 (H3N2) viruses NA activity by CHA. (**B**) The IC_50_ values of CHA against NA activity of both influenza virus strains.

To evaluate the target of CHA against influenza virus, NA activity in the presence of CHA was measured *in vitro*. CHA reduced NA activity of the influenza A/PuertoRico/8/1934 (H1N1) virus and the influenza A/Beijing/32/92 (H3N2) virus in a dose-dependent manner (Fig. 3A), with an IC_50_ of 22.13 ± 1.07 μM against H1N1 and of 59.08 ± 1.12 μM against H3N2 (Fig. 3B). These results demonstrated that CHA inhibited NA and further lead to blocking of release and spread of progeny virions from infected cells, which explained the prevention of CHA on the post-adsorption step of viral life cycle. Thus, inhibitory on NA could be the anti-influenza virus mechanism of CHA.

### Inhibitory effects of CHA on the influenza virus *in vivo*

First, the safety of CHA treatment in mice was assessed. Animals treated with CHA at a dose of 100 mg/kg/d maintained a relatively steady weight and showed no significant clinical symptoms throughout the study (data not shown).

**Figure 4:**
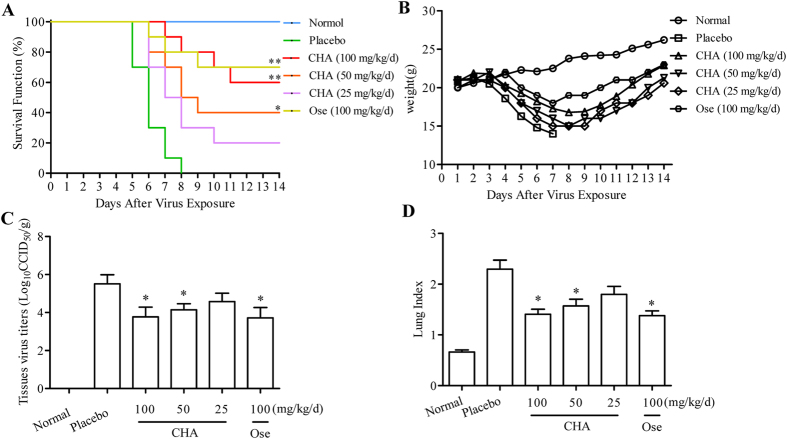
Evaluation of the anti-influenza activity of CHA in H1N1-infected mice. (**A**) Survival rate. Mice were infected with 1 MLD of the influenza A/PuertoRico/8/1934(H1N1) virus and treated with CHA or oseltamivir once a day for 5 days. Clinical signs were observed for 14 days (*n* = 10). Effects of treatment with CHA and oseltamivir on (**B**) the mean body weight during influenza A/PuertoRico/8/1934(H1N1) virus infection (*n* = 10). The mice were killed at 5 d pi. The lungs were removed and rinsed with sterile PBS. The effect of CHA on the (**C**) viral titres in the lungs and the (**D**) lung index of mice were detected (*n* = 5). **P < 0.01 compared to placebo, *P < 0.05 compared to placebo.

To evaluate the therapeutic efficacy of CHA against influenza A/PuertoRico/8/1934 (H1N1) virus, a lethal murine infection model was used. Mice of the placebo group all died at 8 days post infection (dpi). Oseltamivir protected 70% of mice from lethal infection. However, the administration of CHA at 100, 50, and 25 mg/kg/d saved 60%, 40% and 20% of mice infected with H1N1, respectively (Fig. 4A). Administration of CHA effectively protected the infected mice from weight loss caused by influenza virus infection (Fig. 4B). Despite the similar trend of initial weight loss in the first 7 days of infection, animals treated with CHA regained weight starting on day 8, whereas all of the mice without treatment (placebo) showed the most significant weight loss within 8 days. These results demonstrated that CHA treatment effectively increased survival rate and protected mice from weight loss associated with lethal infection with influenza virus.

To further investigate the therapeutic efficacy of CHA against H1N1, virus titres in the lung of mice were determined at 5 dpi. No virus was observed in lung tissues from the normal group. Virus titre of the placebo group was 5.52 ± 0.48 Log_10_CCID_50_/g, whereas virus titres were 3.77 ± 0.51 Log_10_CCID_50_/g and 4.14 ± 0.32 Log_10_CCID_50_/g in the 100 and 50 mg/kg/d CHA-treated groups (Fig. 4C), respectively. H1N1 virus titres were markedly decreased by CHA treatment. The lung index for each group was observed to evaluate lung lesions. The placebo group had a lung index of 2.3 ± 0.31, whereas lung index was 1.41 ± 0.17 and 1.57 ± 0.23 when treated with 100 and 50 mg/kg/d CHA (Fig. 4D), respectively. Therefore, CHA decreased the lung index effectively compared to the placebo group.

**Figure 5:**
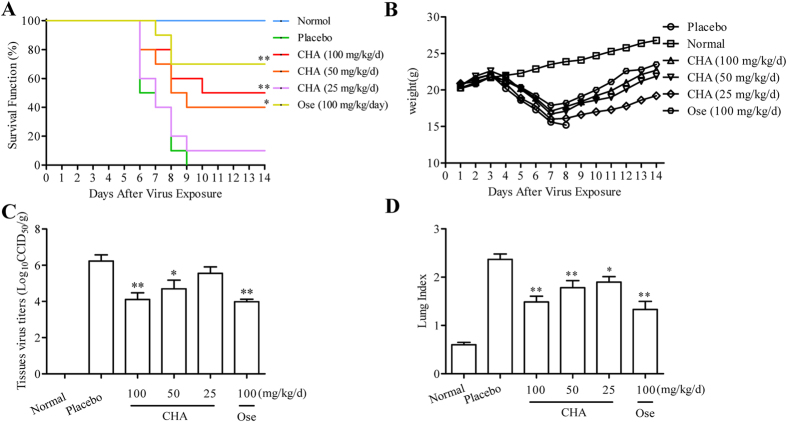
Evaluation of the anti-influenza activity of CHA in H3N2-infected mice. (**A**) Survival rate. Mice were infected with 1 MLD of the influenza A/Beijing/32/92 (H3N2) virus and treated with CHA or oseltamivir once a day for 5 days. Ten mice per group were observed for 14 days for clinical signs of infection or death (*n* = 10). Effects of treatment with CHA and oseltamivir on (**B**) the mean body weight during influenza A/Beijing/32/92 (H3N2) virus infection (*n* = 10). The mice were killed at 5 d pi. The lungs were removed and rinsed with sterile PBS. The effect of CHA on the (**C**) viral titres of the lungs and the (**D**) lung index of mice infected by the influenza A/Beijing/32/92 (H3N2) virus (*n* = 5). **P < 0.01 compared to placebo, *P < 0.05 compared to placebo.

Similar results were required in an H3N2-infected model, CHA treatments of 100 and 50 mg/kg/d resulted in 50% and 40% survival rate (Fig. 5A), protecting infected mice from weight loss (Fig. 5B). Lung virus titers (Fig. 5C) and lung index (Fig. 5D) on day 5 of H3N2-infected mice were significantly reduced by CHA. These results indicated CHA could exhibit anti-influenza activity against H3N2 *in vivo* as well.

### Effects of CHA on H1N1 infection in the lung

**Figure 6:**
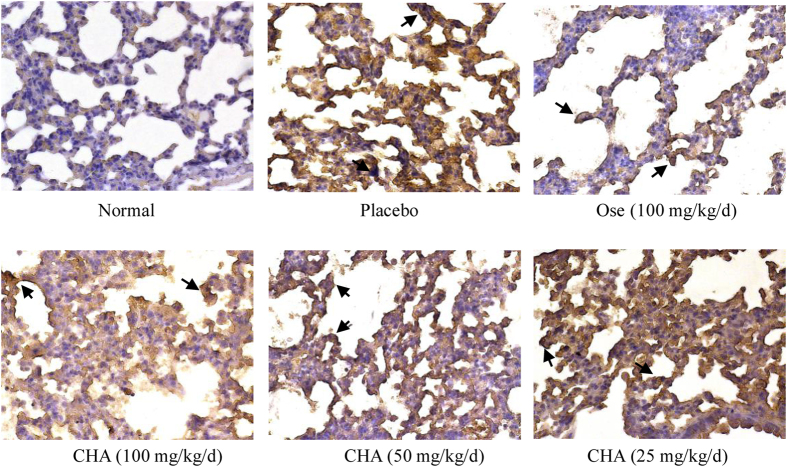
CHA reduced viral antigen in lung tissues from mice infected with H1N1 isolates. Lungs were sectioned for immunohistochemical staining to detect viral NP. Arrows marked in sections of placebo group show a large number of viral NP, while viral NP reduced in CHA-treated group. All micrographs were taken at ×200 magnification.

The expression of NP, which represents virus load in the lung, was detected by immunohistochemistry. Lung sections obtained from the placebo group had the most obvious immunostaining of viral antigens. However, less immunostaining was detected in the CHA-treated group, which could be attributed to the inhibition of viral replication by CHA in the lungs (Fig. 6). These data were also consistent with the results of virus titres in lung tissues.

**Figure 7:**
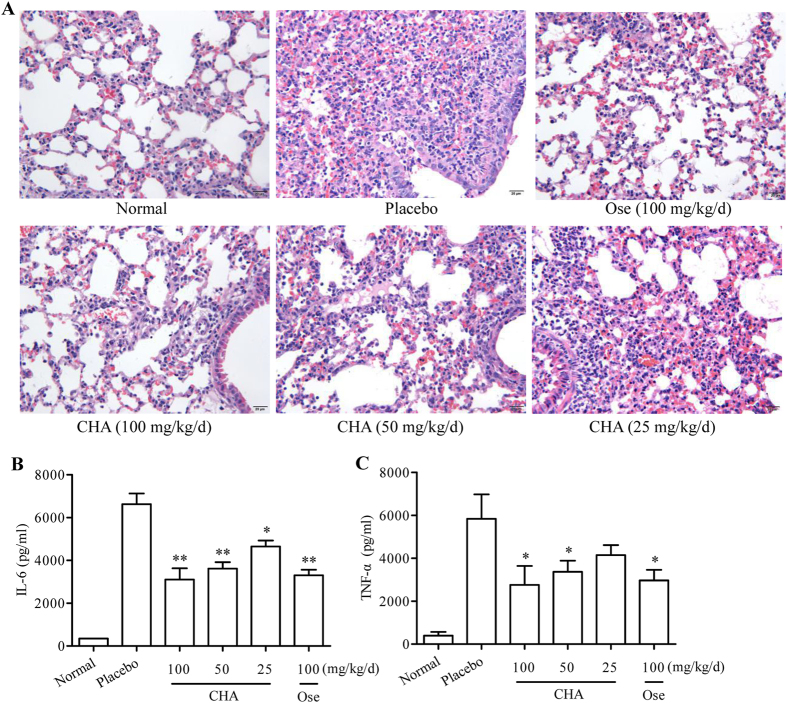
Pathological changes in the lungs of mice infected with the A/PuertoRico/8/1934(H1N1) virus. The mice were killed at 5 dpi. The lungs were removed and rinsed with sterile PBS. (**A**) After being fixed in 10% formalin, the lungs were sectioned for hematoxylin-eosin staining to evaluate pathological injury (*n* = 5). All micrographs were taken at × 400 magnification. Concentrations of IL-6 (**B**) and TNF-α (**C**) in the BAL fluid from mice of each group (n = 5). **P < 0.01 compared to placebo, *P < 0.05 compared to placebo.

The lungs of mice were sampled for histopathologic changes caused by viral infection at 5 d pi by haematoxylin and eosin staining (Fig. 7A). No signs of lung inflammation or pathological changes were observed in the normal control group. Bronchial epithelial cells were necrotic in mice from the placebo group with thickened alveolar walls. Severe lung hyperemia and lesions were observed in the placebo group. Meanwhile, alveolar spaces filled with moderate inflammatory infiltrates of neutrophils, macrophages, and lymphocytes. However, pathological sections of the CHA-treated groups show remission of lung hyperemia and lesions. And the lungs of mice treated with CHA had a reduced inflammatory response, which were consistent with the results of measuring lung index.

To further determine if CHA regulates the secretion of cytokines, bronchoalveolar lavage (BAL) fluids from each group were assessed at 5 d pi. Influenza virus infection resulted in significant IL-6 and TNF-α accumulation in BAL fluid compared with the normal control group, which was clearly reduced by CHA at 5 dpi (Fig. 7B,C). Additionally, the effect of IL-6 down-regulation was more pronounced than that of TNF-α.

## Discussion

Influenza is a highly contagious disease with high morbidity and mortality during an epidemic. The clinical application of anti-influenza drugs is limited by side effects and the emergence of resistant strains^8^. Consequently, it’s very necessary to explore new drugs for influenza virus control. Traditional Chinese medicinal herbs may be a potential alternative medicine source for treatment^31^. Recently, clinical trials have shown that TCMs, including *Lonicera japonica* Thunb, could be alternative treatments for influenza^32^. CHA exists in high quantities in *Lonicera japonica* Thunb^33^, which is an inexpensive and widely distributed resource. Thus, CHA could be used for potential anti-influenza therapy at low cost in light of the continuous emergence of new and virulent influenza strains. Here, the inhibitory effects of CHA against influenza A/PuertoRico/8/1934(H1N1) and A/Beijing/32/92 (H3N2) viruses were investigated both *in vitro* and *in vivo*.

First, the EC_50_ of CHA against H1N1 and H3N2 were 44.87 μM and 62.33 μM *in vitro*, respectively. Moreover, CHA inhibited several oseltamivir-resistant strains, which implied the binding mode of CHA differs from oseltamivir. Thus, CHA, with a board anti-influenza spectrum, could be an alternative therapeutic approach against resistance. *NP* transcription and protein synthesis were significantly decreased by CHA administration, attributed to its antiviral effects. Furthermore, 100 mg/kg/d CHA possessed effective antiviral activity *in vivo*, conferring 60% and 50% protection from death against H1N1 and H3N2. CHA also prolonged survival time, decreased virus titres in the lung, and inhibited lung consolidation in virus-infected mice. These data show a lower dosage than those of previous reports^23^. Oral administration of 960 mg/kg/d CHA caused a survival rate of 56%, in comparison with model group^23^, which could be attributed to the low oral bioavailability (0.13%) of CHA^29^. Thus, it was reasonable that intravenous injection of 100 mg/kg/d CHA caused a similar inhibition of influenza with oral administration of 960 mg/kg/d CHA. Moreover, intravenous injection of CHA could be an optional administration route. Taken together, the antiviral effects of CHA against influenza virus were demonstrated in this study, which agrees with findings of previous studies^17^,^23^,^24^,^25^,^26^. Importantly, CHA could be the potential antiviral material basis of *Lonicera japonica* Thunb^34^, Reduning^35^ and Shuanghuanglian injection^36^.

Greater activity against influenza virus was observed when CHA was added after viral adsorption, which could be attributed to the inhibition of CHA on NA activity, playing a vital role in the viral life cycle with respect to release of progeny virions from infected cells. Indeed, the IC_50_ of CHA against NA of H1N1 and H3N2 was 22.13 μM and 59.08 μM, respectively. Hence, CHA may inhibit the release and spread of progeny virus particles. Interestingly, CHA had a greater inhibitory effect against NA from H1N1 virus than against that from H3N2, which is consistent with the results observed in cellular model. Amino acid differs in or near the active site of NA between two strains which may have effects on inhibitor binding. These differences in the NA amino acid sequence may lead to different structure and thereafter susceptibility to CHA^37^. These results demonstrated that NA could be a potential antiviral target of CHA to counter influenza A virus.

Monocytes and macrophages are susceptible to influenza A virus infection^38^,^39^. In response to excessive viral load, these cells produce cytokines, such as IL-6 and TNF-α^39^. Accumulation of IL-6 and TNF-α is responsible for the pathogenesis and severity of influenza virus infection^40^,^41^, for it could cause severe secondary pneumonia in the lung, which is one of the most important causes of mortality in influenza infection^39^,^42^. In this study, CHA was shown to decrease secretion of IL-6 and TNF-α induced by influenza virus infection, and thereby alleviated inflammation and damage in lung tissues^43^. Thus, the down-regulation of cytokine secretion could be attributed to the inhibition of virus budding caused by CHA. Thus, we conclude that CHA reduced inflammation by inhibiting the excessive secretion of IL-6 and TNF-α in the lung tissue of infected mice.

In summary, this study demonstrates the activity of CHA, as a NA inhibitor, countering influenza A virus infection in both cell culture and mice. Inhibition of NA by CHA decreased virus titres and alleviated inflammation in infected mouse lung tissues. These results suggest that CHA exhibits potential utility in the control of influenza virus infections with limited toxicity.

## Materials and Methods

### Compounds

CHA with the purity of 98% was purchased from the China Pharmacy Biological Products Examination Institute. Oseltamivir carboxylate was purchased from Chembest Co., Ltd. (Shanghai, China).

### Viruses and cells

The influenza strains A/PuertoRico/8/1934(H1N1), A/FM1/1/47 (H1N1), A/Beijing/32/92 (H3N2), and A/Human/Hubei/3/2005(H3N2) were obtained from Wuhan Institute of Virology, China Academic of Sciences. The clinical isolated strains of A/Jinnan/15/2009(H1N1) and A/Zhuhui/1222/2010(H3N2), resistant to oseltamivir and amantadine, respectively, were kindly donated by the Institute for Viral Disease Control and Prevention, China Center for Disease Control and Prevention, and stored at −80 °C. Madin-Darby canine kidney (MDCK) cells were purchased from the American Type Culture Collection (ATCC, Manassas, VA, USA). 1640 medium supplemented with 10% (V/V) FBS, 100 U/ml penicillin and 100 U/ml streptomycin was used for culturing cells at 37 °C in a humidified atmosphere of 5% CO_2_.

### Animals

Specific-pathogen-free BALB/c mice 6 weeks of age and weighing 18–22 g were purchased from the Animal Experimental Centre, Yangzhou University, China (No. SCXK (Jiangsu) 2012–0004)^44^. Animals were housed in a 12 h light/dark cycle, and the air temperature was maintained at 22 ± 2 °C. This study was carried out in strict accordance with the recommendations in the Guide for the Care and Use of Laboratory Animals of the National Institutes of Health. All animal experiments protocols were approved by Laboratory Animal Association of Jiangsu (Licence number: SYXK(Jiangsu)2010–0010), which were conducted in accordance with the “Guiding Opinions on PETA’s” promulgated by Ministry of Science and Technology of China in 2006.

### Cytotoxicity assay

An MTS assay was performed to evaluate the cytotoxic effects of CHA on MDCK cells. A series of concentrations of CHA (0–1000 μM) was added to the cells. After incubation at 37 °C for 48 h, medium with 10% MTS (3-(4,5-dimethylthiazol-2-yl) -5-(3-carboxymethoxyphenyl)-2-(4-sulfophenyl)-2H-tetrazolium) stock solution was added to each well^45^. After 2 h of incubation under culture conditions, absorbance (A490 nm) was measured using a microplate reader, and cell viability was expressed as the percentage of the absorbance value determined with respect to control cultures^7^. The half-maximum cytotoxic concentration (CC_50_) was defined as the concentration that reduced the OD_490_ of CHA-treated cells to 50% of that of untreated cells.

### Antiviral activity assay

An MTS assay was performed to evaluate the antiviral activity of CHA against influenza A viruses. MDCK cells were inoculated with 100 CCID_50_ (50% cell culture infective dose) of different strains of influenza virus suspension in 1640 medium for 2 h at 35 °C. Culture growth medium containing different concentrations of CHA ranging from 5 to 100 μM was added to cells in a confluent monolayer. Oseltamivir carboxylate (2 μM) was used as positive control. All cultures were incubated at 37 °C for 48 h. All wells were then observed under a light microscope to determine CPE^46^,^47^. Inhibition rate (%) = [(mean optical density of test - mean optical density of virus controls)/(mean optical density of cell controls - mean optical density of virus controls)] × 100%. The 50% effective concentration (EC_50_) was calculated using regression analysis, and the selectivity index (SI) was defined as the ratio of CC_50_ to EC_50_.

### Inhibitory effects of CHA on different stages of viral replication

To investigate the anti-influenza effects of CHA at different stages of replication, CHA (10, 50, or 100 μM) were added exclusively for a 12 h pre-incubation period prior to infection (protocol 1), added together with virus for 2 h during adsorption period (protocol 2), or added immediately after the virus adsorption period (protocol 3). Cells were incubated for 48 h at 37 °C and cell viability was detected using an MTS assay.

### Indirect immunofluorescence assay (IFA)

MDCK cells were infected with influenza A/PuertoRico/8/1934(H1N1) virus, after removing influenza virus and washing with PBS, the cells were incubated with several concentrations of CHA (10, 50, 100 μM) diluted in growth medium containing 0.5% FBS. Twenty-four hours pi, the cells were fixed with 4% paraformaldehyde for 30 min. Cells were permeabilized with 0.1% Triton-X100 for 5 min. After blocking with 2% bovine serum albumin for 20 min, the cells were exposed to a FITC-conjugated mouse monoclonal antibody against influenza A virus nucleoprotein (NP) (Merck Millipore, Germany) in 4 °C for 12 h. Cell nuclei were stained with 4′,6-diamidino-2-phenylindole (DAPI), and the cells were visualized under a fluorescence microscope^48^,^49^.

### NA inhibition assay

A fluorescence-based NA inhibition assay was used to determine the sensitivity of the influenza viruses to CHA. The assay is based on the release of a 4-methylumbelliferone fluorescent product from the 2′-(4-Methylumbelliferyl)-α-D-N-acetylneuraminic acid sodium salt hydrate (MU-NANA) (Sigma-Aldrich Co., St. Louis, MO, USA) substrate as a measure of NA activity^7^. The allantoic fluid of embryonated chicken eggs infected with influenza A viruses, containing NA of viruses, was used as enzymatic resources. For the NA inhibition assay, 60 μl of a NA solution was first incubated with 10 μl of CHA (5–100 μM) at 37 °C in black 96-well microplate for 10 min. Next, 30 μl of an 80 μM MU-NANA substrate solution was added, and the plates were incubated at 37 °C for 30 min. Fluorescence was measured at Ex = 355 nm and Em = 460 nm^50^. The IC_50_ values of CHA represent concentrations that caused 50% loss of enzyme activity.

### Antiviral study in mice

Seventy six-week-old female BALB/c mice were grouped into 7 groups: normal control (mice without viral infection); placebo (infected mice without treatment); CHA-treated (25, 50 and 100 mg/kg/d); and oseltamivir-treated (100 mg/kg/d). Oseltamivir treatment was used as a positive control. All mice, except for the normal control group, were anesthetized by ether and intranasally infected with minimum lethal dose (1MLD) of influenza A/PuertoRico/8/1934(H1N1) virus diluted in PBS and then divided randomly into experimental and placebo groups^51^. At 2 hpi, mice were then administrated with CHA by intravenous injection or oseltamivir by oral gavage daily for 5 days. For the normal and placebo group, the mice were given saline water instead. Survival was observed and the mice were weighed daily for 14 days^50^,^52^.

Mice were weighed and euthanized at 5 dpi, and the lungs were removed and weighed^53^. The lung index was calculated as follows using the obtained values: Lung index = A/B × 100, where A is the lung weight, and B is the body weight^54^. Lungs harvested 5 dpi from each group were homogenized in 1640 medium containing antibiotics at 10% w/v tissue. Tissue homogenates, which were first clarified by low-speed centrifugation and then diluted (10^−1^–10^−7^) in 1640 medium, were added in 96-well culture plates containing MDCK cells, and virus titres were expressed as Log_10_CCID_50_/gram tissue. Survival, MDD (Mean day to death), weight, lung index and viral titers were also evaluated in an H3N2 infection in mice treated with CHA.

BAL fluid was collected on 5 dpi by using consecutive instillations of 1 mL PBS. The collected BAL fluid was centrifuged at 1500 rpm at 4 °C for 5 min, and the supernatants were stored at −80 °C^55^,^56^. The concentrations of cytokines IL-6 and TNF-a in the BAL fluid were measured using anti-mouse enzyme-linked immunosorbent assay (ELISA) kits (eBioscience, San Diego, USA) according to the manufacturer’s guidelines.

### Histopathology and immunohistochemical staining

Lungs from each group of mice at 5 dpi were immediately fixed in 10% neutral-buffered formalin, embedded in paraffin wax, and processed for histopathology and immunohistochemical staining. For evaluating influenza viral antigen expression, a monoclonal antibody (Merck Millipore, Germany) against the nucleoprotein of influenza A virus was applied to the sections^2^. Then, the sections were treated with HRP-labelled rabbit anti-mouse IgG(H + L) (Beyotime Biotechnology, China). The slides were visualized using a DAB horseradish peroxidase colour development kit (Beyotime Biotechnology, China). Finally, the slides were observed and photographed under an Olympus light microscope to detect the distribution influenza A virus nucleoprotein^57^,^58^.

### Statistical analyses

All data are expressed as the mean ± standard error (SE). Differences during experiments were analysed by unpaired two-tailed *t*-test.

## Additional Information

**How to cite this article:** Ding, Y. *et al*. Antiviral activity of chlorogenic acid against influenza A (H1N1/H3N2) virus and its inhibition of neuraminidase. *Sci. Rep.* **7**, 45723; doi: 10.1038/srep45723 (2017).

**Publisher's note:** Springer Nature remains neutral with regard to jurisdictional claims in published maps and institutional affiliations.

## Supplementary Material

Supplementary Information

## Figures and Tables

**Table 1:** *In vitro* inhibitory effects of CHA against influenza A/PuertoRico/8/1934(H1N1) virus and influenza A/Beijing/32/92 (H3N2) virus.

Compound	Cell Line	CC_50_^a^ (μM)	A/PuertoRico/8/1934(H1N1)		A/Beijing/32/92 (H3N2)	
			EC_50_^b^ (μM)	SI^c^	EC_50_ (μM)	SI
CHA	MDCK	364.30	44.87	8.12	62.33	5.84
	A549	312.67	38.14	8.20	66.89	4.67

^a^CC_50_ was determined by MTS assay. ^b^EC_50_ was determined by MTS assay. ^c^Ratio of CC_50_ to EC_50_.

**Table 2:** The inhibition of CHA against several stains of influenza virus sensitive or resistant to oseltamivir.

Cell line or virus strains	CHA (μM)		
	CC_50_^a^	EC_50_^b^	SI^c^
MDCK	364.30		
Influenza virus			
A/PuertoRico/8/1934(H1N1)		44.87	8.12
A/FM1/1/47 (H1N1)		39.42	9.24
A/Beijing/32/92 (H3N2)		62.33	5.84
A/Human/Hubei/3/2005(H3N2)		51.23	7.11
A/Jinnan/15/2009(H1N1)^d^		58.34	6.24
A/Zhuhui/1222/2010(H3N2)^d^		71.93	5.06

^a^CC_50_ was determined by MTS assay. ^b^EC_50_ was determined by MTS assay. ^c^Ratio of CC_50_ to EC_50_. ^d^Strains clinically resistant to oseltamivir.
