# Supplementary material for: Antiviral activity of chlorogenic acid against influenza A (H1N1/H3N2) virus and its inhibition of neuraminidase
Source: Sci Rep. 2017 Apr 10;7:45723. doi: 10.1038/srep45723 (PMC5385491; doi:10.1038/srep45723)

Antiviral activity of chlorogenic acid against influenza A (H1N1/H3N2) virus and its inhibition of neuraminidase

Yue Ding, Zeyu Cao, Liang Cao, Gang Ding, Zhenzhong Wang, Wei Xiao*

State Key Laboratory of New-tech for Chinese Medicine Pharmaceutical Process, Jiangsu Kanion Pharmaceutical Co., Ltd., Lianyungang, Jiangsu, China

Results

Inhibitory effect of CHA on NP gene and protein synthesis. To further investigate the inhibitory effects of CHA on viral mRNA expression levels, *NP* mRNA transcript and protein levels were assessed. As illustrated in Suppl. Fig. S1A, CHA suppressed H1N1-induced NP mRNA transcription in MDCK cells by approximately 4-fold at 24 h pi. To measure NP protein levels, we utilized western blot analysis. NP protein expression increased during H1N1 infection at 24 h pi. However, the addition of CHA caused a decrease in NP protein expression (Suppl. Fig. S1B). Therefore, these data indicate that CHA suppresses mRNA transcription and subsequently protein translation during H1N1 infection. These results demonstrate that the administration of CHA inhibits the production of H1N1 in MDCK cells, which supports the findings from our previous time-of-addition experiment.

Materials and Methods

Viral load detection. A qRT-PCR assay was performed to evaluate the inhibition of CHA on the mRNA level of influenza A virus[1]. MDCK cells were infected with A/PuertoRico/8/1934(H1N1) virus (100 $CCID_{50}$), and 100 μL/well of culture growth medium containing different concentrations of CHA (10, 50, or 100 μM) was added to cells in a confluent monolayer. Twenty-four hours post-infection (pi), total RNA was isolated from MDCK cells using the TRIzol reagent (Invitrogen, USA)[2], and cDNA was synthesized using random hexamers with a reverse transcript kit (PrimeScript™ 1st Strand cDNA Synthesis Kit, TaKaRa, China) according to the user manuals. The cDNA was subjected to a H1N1 RNA Detection Kit (Shanghai ZJ Bio-Tech Co., Ltd) specific for the *NP* gene. Positive fragments of *NP* gene adjusted to a series of concentrations was used as the standard curve[3,4].

Western blot analysis. A western blot assay was performed to evaluate the inhibition of CHA on the NP expression level of influenza A virus. MDCK cells were infected with A/PuertoRico/8/1934(H1N1) virus (100 $CCID_{50}$), and 100 μL/well of culture growth medium containing different concentrations of CHA (10, 50, or 100 μM) was added to cells in a confluent

monolayer. Twenty-four hours pi, cells were lysed in RIPA medium buffer (Beyotime Institute of Biotechnology, China). Total protein concentration was determined using the bicinchoninic acid protein assay kit (Beyotime Institute of Biotechnology, China). Samples were subjected to SDS-PAGE using a 12.5% acrylamide resolving gel and were transferred to a PVDF membrane (Millipore, USA). The membrane was blocked with 2% non-fat dried milk solution in Tris-buffered saline containing 0.1% Tween-20 for 2 h and then incubated overnight at 4°C with a primary antibody against Influenza A virus nucleoprotein (NP) (Abcam, UK). Immune complexes were detected using HRP-conjugated rabbit anti-mouse IgG. The expression level of GADPH was utilized as an internal standard. Proteins were visualized by enhanced chemiluminescence using a commercially available kit (Amersham, Freiburg, Germany)[5,6].

References

1. Lee SM, Gai WW, Cheung TK, Peiris JS (2011) Antiviral effect of a selective COX-2 inhibitor on H5N1 infection in vitro. Antiviral Res 91: 330-334.
2. Liu R, An L, Liu G, Li X, Tang W, et al. (2015) Mouse lung slices: An ex vivo model for the evaluation of antiviral and anti-inflammatory agents against influenza viruses. Antiviral Res 120: 101-111.
3. Li SW, Yang TC, Lai CC, Huang SH, Liao JM, et al. (2014) Antiviral activity of aloe-emodin against influenza A virus via galectin-3 up-regulation. Eur J Pharmacol 738: 125-132.
4. Cao Z, Ding Y, Ke Z, Cao L, Li N, et al. (2016) Luteoloside Acts as 3C Protease Inhibitor of Enterovirus 71 In Vitro. PLoS One 11: e0148693.
5. Ortigoza MB, Dibben O, Maamary J, Martinez-Gil L, Leyva-Grado VH, et al. (2012) A novel small molecule inhibitor of influenza A viruses that targets polymerase function and indirectly induces interferon. PLoS Pathog 8: e1002668.
6. Sasaki Y, Kakisaka M, Chutiwitoonchai N, Tajima S, Hikono H, et al. (2014) Identification of a novel multiple kinase inhibitor with potent antiviral activity against influenza virus by reducing viral polymerase activity. Biochem Biophys Res Commun 450: 49-54.

Suppl. Fig 1. CHA inhibits the production of nucleic acid and viral protein. (A) The RNA viral load was determined in MDCK cells infected with influenza A/PuertoRico/8/1934(H1N1) virus at 24 h pi using a real-time PCR kit specific for the *NP* gene. (B) NP was detected by Western blot in MDCK cells infected with influenza A/PuertoRico/8/1934(H1N1) virus 24 h pi. ** *P*<0.01 compared to placebo, * *P*<0.05 compared to placebo

Supplementary Figure S1

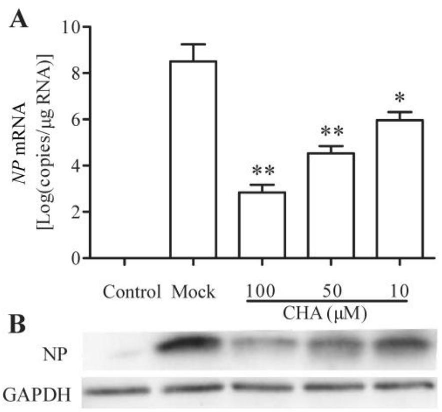

Supplement: Supplementary Information [file srep45723-s1.pdf]
